# Measurement properties of the EQ-5D-5L in sub-health: evidence based on primary health care workers in China

**DOI:** 10.1186/s12955-023-02105-1

**Published:** 2023-03-08

**Authors:** Yueyue Liu, Chuchuan Wan, Xiaoyu Xi

**Affiliations:** The Research Center of National Drug Policy & Ecosystem, China Pharmaceutical University, No.639 Longmian Avenue, Jiangning District, Nanjing, Jiangsu Province China

**Keywords:** EQ-5D-5L, Sub-health, Measurement properties, Validity, Health-related quality of life

## Abstract

**Background:**

Sub-health which is the state between health and disease is a major global public health challenge. As a reversible stage, sub-health can work as a effective tool for the early detection or prevention of chronic disease. The EQ-5D-5L (5L) is a widely used, generic preference-based instrument while its validity in measuring sub-health is not clear. The aim of the study was thus to assess its measurement properties in individuals with sub-health in China.

**Methods:**

The data used were from a nationwide cross-sectional survey conducted among primary health care workers who were selected on the basis of convenience and voluntariness. The questionnaire was composited of 5L, Sub-Health Measurement Scale V1.0 (SHMS V1.0), social-demographic characteristics and a question assessing the presence of disease. Missing values and ceiling effects of 5L were calculated. The convergent validity of 5L utility and VAS scores was tested by assessing their correlations with SHMS V1.0 using Spearman’s correlation coefficient. The known-groups validity of 5L utility and VAS scores was assessed by comparing their values between subgroups defined by SHMS V1.0 scores using the Kruskal–Wallis test. We also did an analysis in subgroups according to different regions of China.

**Results:**

A total of 2063 respondents were included in the analysis. No missing data were observed for the 5L dimensions and only one missing value was for the VAS score. 5L showed strong overall ceiling effects (71.1%). The ceiling effects were slightly weaker on the “pain/discomfort” (82.3%) and “anxiety/depression” (79.5%) dimensions compared with the other three dimensions (nearly 100%). The 5L weakly correlated with SHMS V1.0: the correlation coefficients were mainly between 0.2 and 0.3 for the two scores. 5L was yet not sensitive in distinguishing subgroups of respondents with different levels of sub-health, especially the subgroups with adjacent health status (*p* > 0.05). The results of subgroup analysis were generally consistent with those of the full sample.

**Conclusions:**

It appears that the measurement properties of EQ-5D-5L in individuals with sub-health are not satisfactory in China. We thus should be cautious to use it in the population.

**Supplementary Information:**

The online version contains supplementary material available at 10.1186/s12955-023-02105-1.

## Background

In parallel with the change of living environment and the increasing pace of life, more and more people are in the state of sub-health [1], which refers to the state between health and disease that does not meet the criterion for health nor the clinical diagnostic criteria for diseases according to modern medicine [2]. Chinese scholar Wang Yuxue first formally introduced its concept in the 1980s [3]. It is derived from traditional Chinese medicine (TCM) characterized by a decline in vitality, physiological function and the capacity for adaptation over a certain period of time [2, 4]. In many countries, much attention has been paid on perceived poor health “medically unexplained symptoms (MUS)” [5]. They are two concepts that have some similarities but are not exactly identical. MUS contains a series of clinical defined conditions commonly with diagnostic criteria mainly focusing on physiological symptomatic outcomes like pain and fatigue [6, 7]. However, sub-health is not a strict clinical concept and should be considered as a dynamic process. It includes physical, mental and social adaptation performance of the subject, which highlights both psychological and social factors, so it carries a wider connotation than MUS [3, 8, 9].

Sub-health now is a major global public health challenge [6, 7, 10, 11]. Previous investigations conducted in China within different groups of people have shown that 60%-70% of surveyed individuals are sub-healthy [12, 13]. Sub-health is a low-quality health state and people in this state are typified by impaired health related quality of life (HRQoL) [6, 7, 10]. They may frequently suffer from physical, mental and social interactional problems, like fatigue, pain, sleep disorder, depression, agitation, fear, inability to assume appropriate social roles and so on. As a reversible stage, sub-health has a bidirectional transformation to health or disease so it can work as a effective tool for the early detection or prevention of chronic disease [4, 14–17]. Hence, it is necessary to accurately measure sub-health status, which can help to promote early interventions in the population and thus to avoid the generation of disease and further to reduce the burden of disease and healthcare expenditure.

Sub-health is rich in connotation and should be comprehensively assessed from various aspects. Currently the assessment and measurement of sub-health is based on individual symptoms and social background, physiological and biochemical test results, relevant TCM theories, specific questionnaires and scales, or a combination of those methods [18]. However, there is no standardized criterion. Instrument-based measurement can reflect the subjective and multidimensional manifestations of sub-health in a relatively objective manner, which is a widely used sub-health measurement method. Its advantages include being quantitative and easy-to-use. The instruments used in current sub-health researches include Sub-Health Measurement Scale V1.0 (SHMS V1.0) [19], Suboptimal Health Status Questionnaire-25 (SHSQ-25) [20, 21], Sub-Health Self-Rating Scale (SHSRS) [22], Self-Rated Health Measurement Scale Version1.0 (SRHMS V1.0) [23, 24], etc. Among them, the SHMS V1.0 developed by Xu et al. is a reliable and valid tool widely used for measuring sub-health (Cronbach's alpha coefficient of 0.917 and KMO statistic of 0.927) [1, 12, 13, 19, 25–29]. Although those scales can reflect the health status of individuals, the variety of them hinders the result comparisons between studies using different scales due to not exactly consistent development ideas behind those scales. Moreover, the instruments cannot provide health utilities, thus cannot be used in economic evaluations. The EQ-5D-5L (5L) is a widely used, generic preference-based instrument with good reliability and validity in many specific groups of people [30–32]. If it is a valid sub-health measurement instrument, health utilities in sub-health and result comparisons among different studies could be available, which lay a foundation for further researches. However, to the best of our knowledge, there is a lack of study on the performance of 5L in sub-healthy population at present.

Hence, the study aimed to assess whether the 5L can effectively reflect the impact of sub-health on HRQoL using the SHMS V1.0 as an external standard. Given the fact that primary health care workers in China are at high risk of being sub-health due to their long-term heavy workloads [33], the study was conducted based on data from primary health care workers in China.

## Methods

### Sampling and data collection

The study used data from a cross-sectional survey conducted in primary health care institutions across multiple cities in mainland China from July to August 2021. The definition of primary health care institutions in this survey refers to the relevant provisions of *Law of the people’s Republic of China on the Promotion of Basic Medical and Health Care*. They mainly include township (street) health centers, community health service centers (stations), village health offices, infirmaries and clinics. Respondents meeting the following criteria were included in the analysis: (1) Full-time health care workers of the institutions; (2) ≧1 year of working experience; (3) Not clinically diagnosed with any disease.

The study sample was selected on the basis of convenience and voluntariness. First, thirty one provinces, autonomous regions and municipalities in mainland China were divided into four region groups (ten in the Eastern Region, six in the Central Region, twelve in the Western Region, and three in the Northeast Region) according to their economic levels and future development strategies. Then investigators from all provinces, autonomous regions and municipalities included in each region were recruited respectively. After receiving uniform training, investigators visited at least two local primary care institutions by convenience to conduct face-to-face questionnaire survey. At the study site, according to the principle of voluntariness, investigators invited health care workers to participate in the survey, providing them with the purposes, contents, and requirements of the survey. Then, investigators confirmed with consenting individuals the time and undisturbed places for the survey. After being reminded of the bullet points and prompts in questions, the respondents answered questionnaires independently on removable electronic devices (mobile phone, tablet, etc.) provided by the investigators, and the data were uploaded to the electronic database system in real time. We assigned data auditors to examine the collected data in time. If many missing items or logical errors were found, the questionnaire will be discarded and fed back to the corresponding investigator so as to timely conduct the investigation again. The questionnaire relevant to this study was composited of social-demographic characteristics (including age, gender, height, weight, education level, years of experience, professional title, annual income, type of household, marital status, region and location of healthcare institution), 5L, SHMS V1.0 and a question assessing the presence of disease. The order of the SHMS V1.0 and 5Lwas randomly assigned to avoid order effect. The two scales’ items were respectively set as 5L and SHMS V1.0 question groups. Then we set up two “element packages” containing the 5L and SHMS V1.0 question sets in reverse order. In each questionnaire, only one “element package” was randomly displayed to realize the randomization of the response order of the two scales.

The protocol of this study was approved by the Ethics Committee of China Pharmaceutical University (No. CPU2019015). Informed consent was obtained from all individual participants included in the study.

### Instruments

#### SHMS V1.0

SHMS V1.0 has been widely used in college students, residents in different regions, and has showed good reliability and validity in measuring sub-health [25–27]. Its Cronbach's alpha coefficient was 0.939 for our sample. SHMS V1.0 is composited of three subscales, ten dimensions and 39 items in total, each in a Likert 5-point format, with four general items of sub-health (GS). The three subscales are physical sub-health scale (PS), mental sub-health scale (MS) and social sub-health scale (SS). PS contains four dimensions of physical symptom, organic function, physical mobility function, and vitality. MS includes three dimensions of positive emotion, psychosocial symptom and cognitive function. SS contains three dimensions of social adaptability, social resource and social support. For SHMS V1.0, the conversion score (s_c_) is adopted to do analysis. It is defined as:

$${\text{s}}_{{\text{c}}} = \frac{{s_{r} - s_{{r\left( {min} \right)}} }}{{s_{{r\left( {max} \right)}} - s_{{r\left( {min} \right)}} }} \times 100,$$

where s_r_ is the raw score calculated by summing the corresponding item scores except ones of four overall assessment items. s_r(min)_ is the theoretical minimum score of s_r_ and s_r(max)_ is the theoretical maximum score of s_r_. Higher conversion scores mean better health status.

#### EQ-5D-5L

5L is a generic preference-based instrument for measuring health and consists of two parts: a descriptive system and a visual analog scale (VAS). The system comprises five dimensions of mobility, self-care, usual activities, pain/discomfort, and anxiety/depression, with each consisting of only one item. Each item has five levels of response describing no, slight, moderate, severe, and extreme problems (“1” being the no problems and “5” being the extreme problems). The 5L can describe a total of 3125 potential health states of the respondent on the day of survey, with “11,111” being the full health and “55,555” the worst health state. EQ-VAS is a vertical line with a scale ranging from 0 to 100, with 100 on the top representing the “best imaginable health state” and 0 at the bottom representing the “worst imaginable health state”. In the study, the responses to the five dimensions of 5L were converted into utility scores using the Chinese 5L value set [34].

### Data analysis

Descriptive statistics including mean, standard deviation (SD) and percentage were used to present characteristics of the study sample. The mean values (SD) of 5L utility and EQ-VAS scores were respectively calculated and the missing data of 5L were examined. The distributions of responses to different dimensions and the proportion of respondents reporting the full health of 5L were also reported.

We assessed the convergent validity of 5L by analyzing its relationship with SHMS V1.0, using Spearman’s correlation coefficient. We hypothesized that the respective correlation of 5L utility and EQ-VAS scores with SHMS V1.0 total and subscale scores would be moderate or strong, as well as the similar dimensions from the two instruments including Pain/discomfort and Organic function; Mobility and Physical mobility function; Pain/discomfort and Physical mobility function; Usual activities and Vitality; Anxiety/depression and Psychosocial symptom. The values of correlation coefficients less than 0.3 were considered to be weak, values between 0.3 and 0.49 were regarded as moderate, and values of 0.5 and above as strong [35].

The known-groups validity of 5L was assessed by identifying whether subgroups known to be different in health status could be distinguished by 5L’s utility and VAS scores [36]. Five known-groups (i.e., health, mild sub-health, moderate sub-health, severe sub-health and illness) were defined according to SHMS V1.0 total, PS, MS or SS score respectively. The demarcation score boundaries were determined according to the demarcation norms of the SHMS V1.0 in Chinese civil servants (Additional file 1) [37]. Since both the distributions of utility and VAS scores were skewed, the non-parametric Kruskal–Wallis H rank test with pairwise comparisons was performed to assess their known-groups validity. We hypothesized that the higher the SHMS V1.0 total, PS, MS or SS scores, the higher the 5L utility and EQ-VAS scores.

Since the responses to preference-based HRQoL scales may vary among different regions [38], we also analyzed the convergent validity and known-groups validity in subgroups determined by different regions of China (i.e., the Eastern, Central, Western and Northeast Regions).

Microsoft® Excel 2016 and IBM SPSS 26 were used for data analysis. All effects were considered statistically significant at p < 0.05*.*

## Results

### Descriptive statistics

A total of 2167 questionnaires from 913 primary health care institutions nationwide were returned and 2063 respondents (34.7% in the Eastern Region, 25.8% in the Central Region, 31.8% in the Western Region, and 7.7% in the Northeast Region) were included in the final analysis, with the response rate being 95.2%.

Table 1 displays characteristics of the study respondents. Six hundred and thirty-four (30.7%) were male and 1429 (69.3%) were female. Their mean age (SD) was 37.6 (9.6) years.

**Table 1:** Characteristics of the study respondents

	n (%)
*Gender*	
Male	634 (30.7)
Female	1429 (69.3)
*Age (years)*	
Mean ± SD^a^	37.6 ± 9.6
< 35	833 (40.4)
[35, 60)	1195 (57.9)
≥ 60	35 (1.7)
*Body mass index* (kg/m^2^)	
< 18.5	193 (9.4)
[18.5, 24)	1403 (68.0)
[24, 28)	408 (19.8)
[28, 30)	19 (0.9)
≥ 30	40 (1.9)
*Education*	
Secondary school or lower	185 (9.0)
College degree	837 (40.6)
Bachelor’s degree	896 (43.4)
Master's degree	136 (6.6)
PhD degree	9 (0.4)
*Years of experience (years)*	
< 5	416 (20.2)
[5, 10)	477 (23.1)
[10, 20)	628 (30.4)
≥ 20	542 (26.3)
*Professional title*	
Junior title	1149 (55.7)
Intermediate title	673 (32.6)
Associate senior title	138 (6.7)
Senior title	46 (2.2)
No title	57 (2.8)
*Annual income level (RMB)*	
< 50,000	651 (31.6)
[50,000, 100,000)	928 (45.0)
[100,000, 200,000)	417 (20.2)
≥ 200,000	67 (3.2)
*Household type*	
Agricultural	764 (37.0)
Non-agricultural	1299 (63.0)
*Marital status*	
Single	368 (17.8)
Married	1676 (81.2)
Others	19 (0.9)
*Region*	
Eastern Region	716 (34.7)
Central Region	532 (25.8)
Western Region	657 (31.8)
Northeast Region	158 (7.7)
*Location of healthcare institute*	
City	1114 (54.0)
Rural area	949 (46.0)

^a^Standard deviation

There were no missing responses to the 5L dimensions and only one missing value for the VAS score. The mean values (SD) of 5L utility and VAS scores were 0.974 (0.057) and 86.4 (14.9), respectively. A total of 1466 (71.1%) respondents reported full health (“11,111”). The distributions of the responses to 5L are shown in Table 2. Nearly all respondents reported no problems on the mobility, self-care and usual activities dimensions. Around 80% reported no problems on the dimensions "pain/discomfort" and "anxiety/depression", and nearly 20% reported slight problems (Level 2). Almost no respondents reported serious or extreme problems on all of the dimensions (Level 4 and Level 5).

**Table 2:** Distributions of the responses to the EQ-5D-5L dimensions

EQ-5D-5L dimension	Level1 (%)	Level2 (%)	Level3 (%)	Level4 (%)	Level5 (%)
Mobility	2020 (97.9)	38 (1.8)	3 (0.1)	0 (0.0)	2 (0.1)
Self-care	2056 (99.7)	3 (0.1)	1 (0.0)	0 (0.0)	3 (0.1)
Usual activities	2029 (98.4)	32 (1.6)	1 (0.0)	0 (0.0)	1 (0.0)
Pain/discomfort	1698 (82.3)	344 (16.7)	19 (0.9)	1 (0.0)	1 (0.0)
Anxiety/depression	1641 (79.5)	386 (18.7)	33 (1.6)	2 (0.1)	1 (0.0)

### Convergent validity

The 5L utility score weakly correlated with SHMS V1.0 scores. Comparing the correlations of 5L utility score with SHMS V1.0 total and the three subscales scores, the 5L utility score most strongly correlated with the SHMS V1.0 total score and weakliest related to the SS score. The correlations between the EQ-VAS and SHMS V1.0 scores were similar but they were generally better than those between the 5L utility score and SHMS V1.0 scores (Table 3). The correlation coefficients for the five pairs of potentially relevant dimensions between SHMS V1.0 and 5L were all lower than 0.3 (weak correlation). Correlation coefficient of the SHMS V1.0 vitality dimension and the 5L usual activities dimension was only -0.056 (Table 4).

**Table 3:** Convergent validity of the EQ-5D-5L utility score and EQ-VAS score

	EQ-5D-5L utility score	EQ-VAS score
SHMS V1.0 total score	0.230*	0.307*
SHMS V1.0 PS score	0.227*	0.267*
SHMS V1.0 MS score	0.202*	0.282*
SHMS V1.0 SS score	0.138*	0.219*
EQ-VAS	0.367*	1.000

*p < 0.01

**Table 4:** The correlations between similar dimensions from SHMS V1.0 and EQ-5D-5L

SHMS V1.0	EQ-5D-5L	Spearman’s Correlation coefficient
Organic function (P2)	Pain/discomfort	− 0.185**
Physical mobility function (P3)	Mobility	− 0.099**
Physical mobility function (P3)	Pain/discomfort	− 0.185**
Vitality (P4)	Usual activities	− 0.056*
Psychosocial symptom (M2)	Anxiety/depression	− 0.208**

**p < 0.01, *p < 0.05

The results of the subgroup analysis were generally consistent: correlation coefficients were generally lower than 0.3 (Additional file 2). The correlations of 5L utility score and EQ-VAS with SHMS V1.0 were best respectively in the Central Region and Northeast Region. The 5L weakliest correlated with SHMS V1.0 SS score for all subgroups. The correlations between EQ-VAS and SHMS V1.0 in all regions were slightly better compared with those between the 5L utility score and SHMS V1.0.

### Known-groups validity

The mean values (SD) of 5L utility score and EQ-VAS score for each group are shown in Table 5. The utility and VAS scores of 5L were both significantly different in at least two of the five groups divided by overall, physical, mental or social health status (*p* < 0.001, Table 5). The Kruskal–Wallis H value of the 5L utility score in the physical health was 113.1 which was higher than those in the mental health (87.5) and social health (43.0). The EQ-VAS also had the lowest Kruskal–Wallis H value in social health (101.2) compared with those in physical and mental health (130.2 and 155.9). The 5L utility score did not significantly distinguish the “illness” and “severe sub-health” groups nor the “health” and “mild sub-health” groups in overall, physical, mental and social health (*p* > 0.05, Additional file 3). EQ-VAS was also insensitive in distinguishing between adjacent health status groups. Subgroup analysis had generally consistent results (Additional file 2).

**Table 5:** Known-groups validity of EQ-5D-5L utility score and EQ-VAS score

	n(%)	EQ-5D-5L utility score Mean (SD^a^)/Mean rank	EQ-VAS score Mean (SD^a^)/Mean rank
*Overall health status*			
Health	584 (28.3)	0.986 (0.036)/1158.43	89.9 (13.3)/1235.16
Mild sub-health	514 (24.9)	0.977 (0.075)/1077.72	88.4 (12.0)/1110.39
Moderate sub-health	755 (36.6)	0.969 (0.050)/957.89	84.2 (16.2)/904.39
Severe sub-health	123 (6.0)	0.964 (0.051)/885.49	80.7 (17.3)/787.83
Illness	87 (4.2)	0.937 (0.088)/763.44	77.7 (16.7)/657.77
Kruskal–Wallis H		103.432	170.634
*p* value		0.000	0.000
*Physical health status*			
Health	726 (35.2)	0.981 (0.065)/1122.88	89.2 (12.8)/1180.01
Mild sub-health	528 (25.6)	0.981 (0.044)/1097.71	87.3 (15.1)/1081.64
Moderate sub-health	603 (29.2)	0.969 (0.049)/952.81	85.1 (13.8)/915.57
Severe sub-health	127 (6.2)	0.952 (0.058)/780.91	78.5 (18.2)/703.97
Illness	79 (3.8)	0.938 (0.087)/765.76	77.0 (22.8)/756.00
Kruskal–Wallis H		113.093	130.167
*p* value		0.000	0.000
*Mental health status*			
Health	488 (23.7)	0.986 (0.034)/1154.32	89.8 (13.9)/1241.48
Mild sub-health	438 (21.2)	0.977 (0.077)/1078.23	88.4 (14.0)/1136.45
Moderate sub-health	83 (140.3)	0.972 (0.050)/1001.35	85.3 (14.8)/952.33
Severe sub-health	208 (10.1)	0.963 (0.057)/896.08	81.9 (16.0)/796.18
Illness	98 (4.8)	0.943 (0.078)/764.03	78.9 (16.5)/698.16
Kruskal–Wallis H		87.543	155.853
*p* value		0.000	0.000
*Social health status*			
Health	367 (17.8)	0.982 (0.042)/1123.47	90.1 (12.9)/1237.30
Mild sub-health	510 (24.7)	0.977 (0.048)/1072.76	86.8 (15.2)/1061.11
Moderate sub-health	848 (41.1)	0.974 (0.066)/1019.71	86.6 (14.2)/1020.05
Severe sub-health	211 (10.2)	0.962 (0.062)/901.40	82.0 (15.9)/805.94
Illness	127 (6.2)	0.964 (0.052)/903.02	80.2 (18.1)/777.21
Kruskal–Wallis H		43.000	101.237
*p* value		0.000	0.000

^a^Standard deviation

## Discussion

This is the first study to assess the measurement performance of 5L in measuring the effect of sub-health on HRQoL, filling a gap in the field of the applicability of 5L in sub-healthy population and laying a foundation for further research in sub-health measurement. Based on the results of a representative sample of primary health care workers in China, the measurement properties of 5L in sub-health may not be satisfactory. Many studies have reported that the 5L shows good measurement properties in many types of cancer [30], hemophilia [31], osteoarthritis [32], etc. The health status of people with clinically diagnosed disease usually declines significantly, while the majority (86%) of the respondents in sub-health of this study were mild to moderate, the difference between whose health status and full health was not obvious. The possible reason could be that the 5L, although a new version of EQ-5D, is still insensitive to the health difference between slightly declined health status and full health.

There were no missing responses to the all 5L dimensions and the instrument had good completion rate, which was consistent with the results of several studies before [32, 39, 40], suggesting its good feasibility in the population. On the other hand, around 70% of respondents reported “no problems” on all the 5L dimensions. This result is higher than that of the general population (50.8%) [41]. The possible reason is that the general population includes people with disease, while the primary health care workers in the study are engaged in their daily work without disease. The proportions of subjects reporting “no problems” on the “pain/discomfort” and “anxiety/depression” dimensions were 82.3% and 79.5%, respectively. Compared with the other three dimensions accounting for nearly 100%, the ceiling effects were slightly weaker on those two specific dimensions. Previous studies in specific patient and general populations have also reported that “anxiety/depression” and “pain/discomfort” were two dimensions presenting relatively more frequent problems [42–44]. It also reflected that the mental problems were important factors affecting the health of primary health care workers in China, which was similar to the existing report [33].

The convergent validity of 5L utility score in measuring sub-health was poor. Its correlations with the SHMS V1.0 overall and three subscales scores were low, especially with the SHMS V1.0 SS score. As a sub-health specific scale, SHMS V1.0 covers a wide range of dimensions in the physical, mental and social health; whereas the 5L contains only five dimensions and lacks dimension related to social health. In terms of similar dimensions between the two instruments, the SHMS V1.0 items are profounder and richer, and the measured content is more comprehensive and detailed. Thus it can detect different health status of the population effectively. For example, the SHMS V1.0 “organic function” dimension includes measurements of vision and hearing in addition to measurements of gastrointestinal and head pain or discomfort. In contrast to the 5L “anxiety/depression” dimension consisting of a single item, the SHMS V1.0 “psychosocial symptom” dimension contains 5 items specifically measuring feelings like nervousness, fear, loneliness and so on. Those feelings often accompany or easily exacerbate to anxiety or depression, so the SHMS V1.0 can capture less severe changes in psychological symptoms. We were surprised that a very low correlation between the "mobility" dimension of the 5L and a similar dimension of the SHMS V1.0, "physical mobility function" (r = − 0.099) was identified. The same was true for 5L "usual activity" dimension and SHMS V1.0 "vitality" dimension (r = − 0.056). In addition, the multiple items within a certain dimension facilitate multiple reflections around that dimension by subjects, and thus better reflect the true situation. The correlations between the EQ-VAS and the SHMS V1.0 overall and three subscale scores were not strong but better compared with those between 5L utility score and SHMS V1.0 scores. It indicates to some extent that the EQ-VAS is able to reflect health dimensions that the 5L descriptive system do not include, which corresponds to the prior finding [45].

Based on the Kruskal–Wallis H values and the results of pairwise comparisons of 5L across the overall, physical, mental and social health, the 5L had the poorest known-groups validity in social health and was not sufficiently effective in distinguishing groups of respondents with different levels of sub-health, especially for the respondents with adjacent health status. The known-groups validity of EQ-VAS score was slightly better compared with the 5L utility score. The possible explanation is that the respondents may provide a global assessment of their health on the EQ-VAS, which is not limited to the five dimensions of 5L.

Previous studies have shown that the 5L could hardly reflect the effects of fatigue, interpersonal relationships, sleep, vision, hearing and drug-induced adverse reactions (such as loss of libido and hair loss) on HRQoL [46–50]. Those factors are important for determining whether an individual is in full health or with varying degrees of sub-health. In addition, 5L takes the same day as the recall period which may not be conducive to the measurement of sub-health characterizing as a long-term chronic state.

There are some limitations to the study. Although SHMS V1.0 is a widely used sub-health measurement instrument, choosing it as an external criterion may still be not enough thus influencing the accuracy of sub-health measurement and the assessment of measurement properties of 5L. The value set used to calculate 5L utility score was derived from a previous study based on a sample of urban residents from five cities in China [35], but 37% of the sample in this study was agricultural households, which might have caused potential bias. In addition, because the study was based on a cross-sectional survey, we cannot evaluate the responsiveness of 5L in the sub-healthy population.

## Conclusions

To conclude, it appears that the EQ-5D-5L lacks measurement properties in measuring HRQoL in sub-healthy population in China. Hence, we should be cautious to use it in some sub-healthy groups whose health is close to full health.

## Supplementary Information

**Additional file 1.** Demarcation norms of the SHMS V1.0 total and subscales scores in Chinese civil servants.

**Additional file 2.** Convergent and known-groups validity of 5L utility score and EQ-VAS score in subgroups divided by different regions of China.

**Additional file 3.** Pairwise comparisons of 5L utility score and EQ-VAS score in known groups divided by health status.

## Data Availability

The datasets generated and/or analyzed during the current study are not publicly available due these data will still be used as auxiliary data in our other studies and we do not wish to publish them publicly but they are available from the corresponding author on reasonable request.

## Abbreviations

TCM: Traditional Chinese medicine
MUS: Medically unexplained symptoms
HRQoL: Health related quality of life
SHMS V1.0: Sub-health measurement scale V1.0
GS: General items of sub-health
PS: Physical sub-health scale
MS: Mental sub-health scale
SS: Social sub-health scale
VAS: Visual analog scale
