# Supplementary material for: Measurement properties of the EQ-5D-5L in sub-health: evidence based on primary health care workers in China
Source: Health Qual Life Outcomes. 2023 Mar 8;21:22. doi: 10.1186/s12955-023-02105-1 (PMC9996950; doi:10.1186/s12955-023-02105-1)
Supplement: Supplementary file 1 — Additional file 1. Demarcation norms of the SHMS V1.0 total and subscales scores in Chinese civil servants. [file 12955_2023_2105_MOESM1_ESM.docx]

**Additional file 1**

**Table 1 The demarcation norms of the SHMS V1.0 total score in Chinese civil servants**

| Groups | | Illness | Severe sub-health | Moderate sub-health | Mild sub-health | Health |
| --- | --- | --- | --- | --- | --- | --- |
| Male | <40 years old | [0, 54） | [54, 60) | [60, 73) | [73, 79) | [79, 100] |
|  | ≧40 years old | [0, 55） | [55, 61) | [61, 74) | [74, 80) | [80, 100] |
| Female | <40 years old | [0, 54） | [54, 60) | [60, 72) | [72, 78) | [78, 100] |
|  | ≧40 years old | [0, 54） | [54, 60) | [60, 72) | [72, 78) | [78, 100] |

**Table 2 The demarcation norms of the SHMS V1.0 PS score in Chinese civil servants**

| Groups | | Illness | Severe sub-health | Moderate sub-health | Mild sub-health | Health |
| --- | --- | --- | --- | --- | --- | --- |
| Male | <40 years old | [0, 56） | [56, 62) | [62, 76) | [76, 83) | [83, 100] |
|  | ≧40 years old | [0, 54） | [54, 60) | [60, 74) | [74, 81) | [81, 100] |
| Female | <40 years old | [0, 55） | [55, 62) | [62, 75) | [75, 82) | [82, 100] |
|  | ≧40 years old | [0, 52） | [52, 59) | [59, 72) | [72, 79) | [79, 100] |

**Table 3 The demarcation norms of the SHMS V1.0 MS score in Chinese civil servants**

| Groups | | Illness | Severe sub-health | Moderate sub-health | Mild sub-health | Health |
| --- | --- | --- | --- | --- | --- | --- |
| Male | <40 years old | [0, 52） | [52, 59) | [59, 74) | [74, 81) | [81,100] |
|  | ≧40 years old | [0, 54） | [54, 62) | [62, 76) | [76, 84) | [84,100] |
| Female | <40 years old | [0, 50） | [50, 58) | [58, 72) | [72, 79) | [79,100] |
|  | ≧40 years old | [0, 53） | [53, 60) | [60, 74) | [74, 82) | [82,100] |

**Table 4 The demarcation norms of the SHMS V1.0 SS score in Chinese civil servants**

| Groups | | Illness | Severe sub-health | Moderate sub-health | Mild sub-health | Health |
| --- | --- | --- | --- | --- | --- | --- |
| Male | <40 years old | [0, 48） | [48, 55) | [55, 71) | [71, 78) | [78, 100] |
|  | ≧40 years old | [0, 50） | [50, 58) | [58, 72) | [72, 79) | [79, 100] |
| Female | <40 years old | [0, 50） | [50, 58) | [58, 73) | [73, 81) | [81, 100] |
|  | ≧40 years old | [0, 51） | [51, 58) | [58, 72) | [72, 80) | [80, 100] |
