# Supplementary material for: Measurement properties of the EQ-5D-5L in sub-health: evidence based on primary health care workers in China
Source: Health Qual Life Outcomes. 2023 Mar 8;21:22. doi: 10.1186/s12955-023-02105-1 (PMC9996950; doi:10.1186/s12955-023-02105-1)
Supplement: Supplementary file 3 — Additional file 3. Pairwise comparisons of 5L utility score and EQ-VAS score in known groups divided by health status. [file 12955_2023_2105_MOESM3_ESM.docx]

**Additional file 3**

**Table 1 Pairwise comparisons of EQ-5D-5L utility score and EQ-VAS score in known groups divided by overall health status**

|  | **Different health status groups** | | **Test Statistic** | **Std.Error** | |
| --- | --- | --- | --- | --- | --- |
|  | **I** | **J** |  |  |  |
| **EQ-5D-5L utility score** | Illness | Severe sub-health  Moderate sub-health  Mild sub-health  Health | -122.049  -194.446*  -314.280*  -394.992* | 66.733  53.935  55.226  54.745 |  |
|  | Severe sub-health | Moderate sub-health Mild sub-health  Health | -72.397  -192.231*  -272.942* | 46.320  47.817  47.260 |  |
|  | Moderate sub-health | Moderate sub-health Health | -119.834*  -200.545* | 27.241  26.252 |  |
|  | Mild sub-health | Health | -80.711 | 28.811 |  |
| **EQ-VAS score** | Illness | Severe sub-health  Moderate sub-health Mild sub-health  Health | -130.059  -246.619*  -452.620*  -577.389* | 82.426  66.618  68.212  67.618 |  |
|  | Severe sub-health | Moderate sub-health Mild sub-health  Health | -116.559  -322.561*  -447.330* | 57.212  59.061  58.374 |  |
|  | Moderate sub-health | Mild sub-health  Health | -206.001*  -330.771* | 33.647  32.425 |  |
|  | Mild sub-health | Health | -124.769* | 35.586 |  |

Note: *p<0.01, and the remaining p values were all greater than 0.05 (Significance values have been adjusted by the Bonferroni correction for multiple tests.)

**Table 2 Pairwise comparisons of EQ-5D-5L utility score and EQ-VAS score in known groups divided by physical health status**

|  | **Different health status groups** | | **Test Statistic** | **Std.Error** | |
| --- | --- | --- | --- | --- | --- |
|  | **I** | **J** |  |  |  |
| **EQ-5D-5L utility score** | Illness | Severe sub-health  Moderate sub-health  Mild sub-health  Health | -15.150  -187.049*  -331.954**  -357.119** | 68.260  56.999  57.466  56.437 |  |
|  | Severe sub-health | Moderate sub-health  Mild sub-health  Health | -171.899**  -316.804**  -341.969** | 46.510  47.081  45.819 |  |
|  | Moderate sub-health | Mild sub-health  Health | -144.905**  -170.070** | 28.392  26.247 |  |
|  | Mild sub-health | Health | -25.165 | 27.246 |  |
| **EQ-VAS score** | Illness | Severe sub-health  Moderate sub-health  Mild sub-health  Health | -52.031  -159.570  -325.645**  -424.014** | 84.311  70.402  70.979  69.708 |  |
|  | Severe sub-health | Moderate sub-health Mild sub-health  Health | -211.602**  -377.676**  -476.046** | 57.447  58.153  56.594 |  |
|  | Moderate sub-health | Mild sub-health  Health | -166.074**  -264.444** | 35.069  32.419 |  |
|  | Mild sub-health | Health | -98.370* | 33.654 |  |

Note: **p<0.01, *p<0.05 (Significance values have been adjusted by the Bonferroni correction for multiple tests.)

**Table 3 Pairwise comparisons of EQ-5D-5L utility score and EQ-VAS score in known groups divided by mental health status**

|  | **Different health status groups** | | | **Test Statistic** | **Std.Error** | |
| --- | --- | --- | --- | --- | --- | --- |
|  | **I** | | **J** |  |  |  |
| **EQ-5D-5L utility score** | Illness | Severe sub-health  Moderate sub-health  Mild sub-health  Health | | -131.449  -236.720**  -313.600**  -389.685** | 58.366  50.879  53.233  52.732 |  |
|  | Severe sub-health | Moderate sub-health  Mild sub-health  Health | | -105.271*  -182.151**  -258.236** | 36.934  40.114  39.447 |  |
|  | Moderate sub-health | Mild sub-health  Health | | -76.880  -152.965** | 28.128  27.168 |  |
|  | Mild sub-health | Health | | -76.085 | 31.355 |  |
| **EQ-VAS score** | Illness | Severe sub-health  Moderate sub-health  Mild sub-health  Health | | -98.022  -254.169**  -438.290**  -543.323** | 72.091  62.844  65.751  65.132 |  |
|  | Severe sub-health | Moderate sub-health  Mild sub-health  Health | | -156.146**  -340.268**  -445.301** | 45.619  49.547  48.723 |  |
|  | Moderate sub-health | Mild sub-health  Health | | -184.122**  -289.155** | 34.742  33.557 |  |
|  | Mild sub-health | Health | | -105.033 | 38.728 |  |

Note: **p<0.01, *p<0.05 (Significance values have been adjusted by the Bonferroni correction for multiple tests.)

**Table 4 Pairwise comparisons of EQ-5D-5L utility score and EQ-VAS score in known groups divided by social health status**

|  | **Different health status groups** | | **Test Statistic** | **Std.Error** | |
| --- | --- | --- | --- | --- | --- |
|  | **I** | **J** |  |  |  |
| **EQ-5D-5L utility score** | Illness | Severe sub-health  Moderate sub-health  Mild sub-health  Health | -1.622  -116.691  -169.743**  -220.452** | 53.501  45.326  47.242  49.043 |  |
|  | Severe sub-health | Moderate sub-health  Mild sub-health  Health | -118.312*  -171.365**  -222.073** | 36.648  38.993  41.156 |  |
|  | Moderate sub-health | Mild sub-health  Health | -53.052  -103.761** | 26.694  29.765 |  |
|  | Mild sub-health | Health | -50.709 | 32.608 |  |
| **EQ-VAS score** | Illness | Severe sub-health  Moderate sub-health  Mild sub-health  Health | -28.732  -242.839**  -283.906**  -460.090** | 66.082  55.985  58.351  60.575 |  |
|  | Severe sub-health | Moderate sub-health  Mild sub-health  Health | -214.106**  -255.174**  -431.358** | 45.266  48.162  50.834 |  |
|  | Moderate sub-health | Mild sub-health  Health | -41.068  -217.251** | 32.971  36.764 |  |
|  | Mild sub-health | Health | -176.184** | 40.276 |  |

Note: **p<0.01, *p<0.05 (Significance values have been adjusted by the Bonferroni correction for multiple tests.)
